# Spectral narrowing in rigid macrocycles

**DOI:** 10.1093/nsr/nwaf366

**Published:** 2025-09-03

**Authors:** Jinbei Wei, Yue Wang

**Affiliations:** State Key Laboratory of Supramolecular Structure and Materials, Jilin University, China; State Key Laboratory of Supramolecular Structure and Materials, Jilin University, China

Supramolecular chemistry has emerged as a dominant frontier of international research in the chemical sciences and is widely recognized as a pivotal source of 21st-century innovations. The evolution of supramolecular chemistry is fundamentally intertwined with advances in macrocyclic architectures [1]. Consequently, the design and synthesis of novel functional macrocycles are persistent research priorities that drive breakthroughs in critical technologies.

Recently, narrowband emitters have gained prominence as a strategic solution to the core challenge of blue organic light-emitting diodes (OLEDs: achieving high efficiency and operational longevity simultaneously [2]. Unlike conventional broadband materials, these emitters maintain lower excited-state energy levels at equivalent color coordinates, enabling theoretically superior device efficiency and stability.

**Figure 1.**
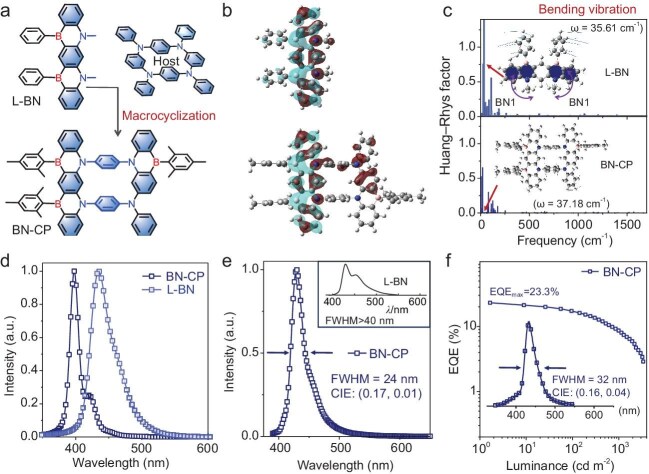
(a) The chemical architectures of L-BN and BN-CP. (b) Distributions of the highest occupied molecular orbital (HOMO, wine) and lowest unoccupied molecular orbital (LUMO, cyan) in L-BN and BN-CP. (c) Huang–Rhys factors under different vibrational modes of L-BN and BN-CP. (d) Simulated photoluminescence spectra of BN-CP and L-BN. (e) Fluorescence spectrum of BN-CP in dilute toluene solution (10^−^^5^ M). Inset: fluorescence spectrum of L-BN in cyclohexane at 298 K. Reproduced from ref. [3] with permission. (f) EQE–luminance (EQE–L) plots based on BN-CP. Inset: electroluminescent spectrum.

Against this backdrop, Duan and Zhang *et al.* demonstrated the superiority of macrocyclization for constructing narrowband deep-blue emitters through an elegant proof-of-concept study [3]. In this design, the researchers embedded a broadband [1,4]azaborine chromophore within a macrocyclic framework (Fig. 1a). By exploiting the inherent rigid architecture of the macrocycle to inhibit vibrational relaxation of the guest chromophore, they achieved dramatic emission bandwidth compression.

Theoretical studies confirm near-identical frontier orbital distributions and vibrational modes in the macrocycle BN-CP and its broadband guest L-BN [3], as shown in Fig. 1b and c. This validates that macrocyclization preserves both the luminescent core architecture and fundamental vibrational modes of the guest chromophore. Consequently, the spectral narrowing in BN-CP directly results from enhanced structural rigidity. This is evident in the significant suppression of L-BN's major vibrational modes, particularly the dominant spectral-broadening bending vibrations of the planes containing the two minimal [1,4]azaborine units, upon macrocyclization (Fig. 1c and d).

As anticipated, BN-CP exhibits an ultra-narrowband deep-blue emission peaking at 430 nm with a substantially

reduced full width at half maximum (FWHM) of only 24 nm compared to the previously documented broadband guest L-BN (Fig. 1e). Leveraging this exceptional spectral purity and high photoluminescence quantum yield (97%) enabled by macrocyclic confinement [5], the BN-CP-based deep-blue OLED achieved a maximum external quantum efficiency (EQE) of 23.3% (Fig. 1f). This EQE performance ranks among the highest values for deep-blue OLEDs at Commission International de l'Eclairage coordinate (CIEy) of ≤0.04. The significance of this strategy extends beyond the material's exceptional performance metrics. As wide-color-gamut displays and 3D visualization technologies become inevitable trends, narrowband macrocyclic luminophores are poised to serve as fundamental building blocks for next-generation OLED devices. This phenomenon can be traced back to their fundamental design principle, which posits that the integration of macrocyclic hosts with (narrowband) luminescent guests facilitates the effective maintenance of single (chiral) conformations over extended spatial distances and concentration ranges. This integration process endows macrocyclic products with superior optoelectronic properties—including anti-aggregation quenching, high color purity, enhanced asymmetric luminescence factors and large horizontal coupling dipole orientation—surpassing those of conventional organic small molecules.
